# Migrant and refugee health: Complex health associations among diverse contexts call for tailored and rights-based solutions

**DOI:** 10.1371/journal.pmed.1003105

**Published:** 2020-03-31

**Authors:** Paul Spiegel, Kolitha Wickramage, Terry McGovern

**Affiliations:** 1 Department of International Health and Center for Humanitarian Health, Johns Hopkins Bloomberg School of Public Health, Baltimore, Maryland, United States of America; 2 Migration Health Division, The UN Migration Agency, Manila, Philippines; 3 Global Health Justice and Governance, Columbia University Mailman School of Public Health, New York City, New York, United States of America

## Abstract

In an Editorial, Guest Editors Paul Spiegel, Terry McGovern and Kol Wickramage discuss the Special Issue on Refugee and Migrant Health.

Migration is a natural state of humankind and has been documented throughout history. Some people may flee violence and persecution, while others simply seek a better life. Although migration is often classified into these two basic categories, the reality is more complex and nuanced: people migrate for a myriad of interconnected cultural, economic, religious, ethnic, and political reasons. Depending upon the epoch, migration has been seen in a positive or a negative light. Currently, the terms migrant and refugee have become politically charged and are widely misused for political and populist purposes. However, no matter how migration is portrayed at a specific point in time, it will inexorably continue. Thus, the need to ensure the protection, health, and welfare of people on the move is imperative and provides the rationale for the accompanying *PLOS Medicine* Special Issue on Refugee and Migrant Health [1]. This imperative is not only a matter of humanity and equity but is also necessary for the global economy, as migration is inherently linked to economic growth [2].

The governance needed to provide health services to this diverse and widespread group of people—from low-waged migrant workers and undocumented migrants to refugees—is unclear. How can we attain universal health coverage in this complex and uncertain environment? At the 72nd World Health Assembly in May 2019, a global action plan was agreed upon that seeks to establish a “framework of priorities and guiding principles…to promote the health of refugees and migrants.” The Global Compact on Migration, developed through intergovernmental negotiations and adopted in December 2018, enshrined health as a cross-cutting priority for migration governance. It is, however, unlikely that governments will apply such frameworks, unfortunately. Despite widespread recognition of the numerous migration-related health risks, mobile populations are often met with punitive border policies, arbitrary detention, abuse, and extortion and are denied access to healthcare. All too often, government policies prioritize the politics of xenophobia over their responsibilities to act forcefully to counter them. As human beings, migrants are entitled to universal human rights without discrimination, and to the “highest attainable standard of health” according to international law. Migration health remains at the margins of policy prioritization for most governments, and thus universal health coverage remains elusive for the vast majority of migrants and refugees [3].

Seeking to raise awareness of the health inequities and different contexts faced by migrants and forcibly displaced persons, as well as to promote research, service, and policy innovation in this area, this Special Issue is devoted to migrant and refugee health in the broadest sense. The articles included, as well as the findings themselves, are as diverse as the topic itself. Here, we discuss the results from some of the articles illustrating different themes to portray this diversity.

The health status of migrants and refugees, along with healthcare coverage and utilization, has quite naturally been explored in some detail among different migrant populations and, unsurprisingly, the health effects vary according to the populations and contexts studied. In a study done in a high-income setting (the city of Bradford in the United Kingdom) where about one-third of mothers had been born in a different country, for example, the proportion of mothers who had visited the emergency department at least once for a consultation involving their children was found to be lower for migrants compared to nonmigrant mothers. However, among all mothers who utilized emergency services, the utilization rate was significantly higher than that of nonmigrant mothers [4]. Such findings can be useful for planning health provision and identifying possible barriers to attendance.

Mobility and relocation can create substantial vulnerabilities, including an increased risk of sexual violence, human trafficking, and labor exploitation, along with a need for child protection [5]. In conflict-affected settings, migration may coincide with weakened protections from family and social networks that leave people, particularly women and girls, vulnerable to exploitation. In a study by Amber Lalla and colleagues, Oromo and Somali refugee women in the Kakuma Refugee camp in Kenya were found to experience multiple sources of insecurity, including violence and neglect, in all spaces of the refugee camp [6]. Health services, including sexual and reproductive health services, are also often limited. However, a qualitative study done in a humanitarian setting in the Democratic Republic of the Congo documents knowledge of contraceptive methods among adolescent and young women as well as unmet need, indicating that other factors may play a greater role in influencing contraceptive use than displacement [7].

While migration often creates new vulnerabilities, it may also serve as a protective factor for migrants leaving highly disadvantaged contexts. In a comparison of international migrants, internal migrants, and nonmigrants in Bangladesh, Randall Kuhn and colleagues [8] found that people who moved primarily to become guest workers in Gulf Cooperative Countries faced comparable or lower injury and mortality risks compared to those who remained in their country of origin.

Health authorities often cite concerns over communicable diseases in migrant populations, which could be perceived to increase risks of disease transmission. However, there are often insufficient data and misinformation about these risks, and the reality is much more nuanced and context specific. A study of HIV diagnosis and care cascades in Australia found an overall improvement among all persons between 2013 and 2017, while cascades for migrants had larger gaps compared to nonmigrants, particularly among key migrant populations [9]. Investigations among Rohingya refugees in Bangladesh found that, despite multiple vaccination campaigns, immunity gaps still existed among children, particularly for diphtheria and polio [10,11].

These and other research studies featured in this Special Issue address a great diversity of migration trajectories and contexts. The evidence harnessed has highlighted different effects and complex associations between migration and health across different settings, including diverse mobility dynamics across different phases of the migration cycle. Policymakers, practitioners, and researchers need to calibrate national and regional policy and programmatic levers by using the best available evidence for their specific context; clearly there is no “one size fits all” conclusion and recommendations when it comes to migration health. Governments and policymakers must commit to and invest in evidence-informed processes while avoiding perceptions and misinformation.

It is clear from the articles in this Special Issue that much of the research in migration health is generated in high-income countries, with limited research productivity on migrant typologies occurring in low- and middle-income countries. With anticipated increases in the numbers of refugees and migrants in the future, there is a clear call for increased investment and support for health research in settings in which the needs of refugees and migrants are greatest. In addition, the health impacts for the largest populations of migrants who are engaged in low-wage work in precarious contexts remain poorly researched [12]. We hope that the research approaches and evidence featured in this issue will encourage future migration health research to address these evidence and equity gaps for the benefit of the growing and vulnerable populations of refugees and migrants worldwide.
